# Markers of endothelial cell activation are associated with the severity of pulmonary disease in COVID-19

**DOI:** 10.1371/journal.pone.0268296

**Published:** 2022-05-19

**Authors:** William O. Osburn, Kimberly Smith, Lisa Yanek, Nuria Amat-Alcaron, David R. Thiemann, Andrea L. Cox, Thorsten M. Leucker, Charles J. Lowenstein

**Affiliations:** 1 Division of Cardiology, Department of Medicine, Johns Hopkins University School of Medicine, Baltimore, MD, United States of America; 2 Division of General Internal Medicine, Department of Medicine, Johns Hopkins University School of Medicine, Baltimore, MD, United States of America; 3 Division of Infectious Diseases, Department of Medicine, Johns Hopkins University School of Medicine, Baltimore, MD, United States of America; University of Utah, UNITED STATES

## Abstract

Severe coronavirus disease-19 (COVID-19) is characterized by vascular inflammation and thrombosis. We and others have proposed that the inflammatory response to coronavirus infection activates endothelial cells, leading to endothelial release of pro-thrombotic proteins. These mediators can trigger obstruction of the pulmonary microvasculature, leading to worsening oxygenation, acute respiratory distress syndrome, and death. In the current study, we tested the hypothesis that higher levels of biomarkers released from endothelial cells are associated with worse oxygenation in patients with COVID-19. We studied 83 participants aged 18–84 years with COVID-19 admitted to a single center. The severity of pulmonary disease was classified by oxygen requirement, including no oxygen requirement, low-flow oxygen, high-flow nasal cannula oxygen, mechanical ventilation, and death. We measured plasma levels of two proteins released by activated endothelial cells, von Willebrand Factor (VWF) antigen and soluble P-Selectin (sP-Sel), and a biomarker of systemic thrombosis, D-dimer. Additionally, we explored the association of endothelial biomarker levels with the levels of pro-inflammatory cytokine and chemokines, and vascular inflammation biomarkers. We found that levels of VWF, sP-sel, and D-dimer were increased in individuals with more severe COVID-19 pulmonary disease. Biomarkers of endothelial cell activation were also correlated with proinflammatory cytokines and chemokines. Taken together, our data demonstrate increased levels of VWF and sP-selectin are linked to the severity of lung disease in COVID-19 and correlated with biomarkers of inflammation and vascular inflammation. Our data support the concept that COVID-19 is a vascular disease which involves endothelial injury in the context of an inflammatory state.

## Introduction

The Severe Acute Respiratory Syndrome Coronavirus-2 (SARS-CoV-2) causes the disease COVID-19. The clinical spectrum of COVID-19 is broad, ranging from mild COVID-19, which is characterized by an upper respiratory tract infection, to moderate COVID-19 which is characterized by pneumonia, systemic inflammation, and thrombosis, to severe COVID-19 which can progress to multi-organ failure, shock, and death.

Severe COVID-19 is a vascular disease, based on reports of clinical events, autopsy studies, and biomarkers. Patients hospitalized with COVID-19 have rates of venous thrombosis of 10–30%, including deep vein thrombosis and pulmonary embolism [1, 2]. The incidence of arterial thromboembolic events is 2–4%, including myocardial infarction and stroke. Autopsy studies have confirmed vascular inflammation and thrombosis in the lungs and in other organs of patients who died of COVID-19. Particularly striking is the pattern of pulmonary microvascular injury in patients who have died with COVID-19, showing thrombi and inflammatory infiltrates in pulmonary capillaries [3]. Furthermore, COVID-19 leads to elevated inflammatory markers, such as C-reactive protein (CRP), interleukin-6, and tumor necrosis factor alpha (TNF-α). COVID-19 also leads to increased thrombosis biomarkers such as D-dimer and plasminogen activator inhibitor-1 and alpha-2-antiplasmin activity [4]. Taken together, clinical and laboratory evidence suggests that vascular injury plays a role in severe COVID-19.

The pathways of vascular damage in severe COVID-19 are unknown. We and others have proposed that direct or indirect viral injury of vascular endothelial cells triggers endothelial cell exocytosis, a process in which activated endothelial cells rapidly release von Willebrand factor (VWF) and soluble P-selectin (sP-selectin) [5]. VWF and sP-selectin are important mediators of thrombosis and vascular inflammation at sites of vascular injury [1]. VWF mediates platelet adherence to the vessel wall and platelet aggregation with other platelets. sP-selectin mediates leukocyte adherence to the vessel wall, the first step in leukocyte trafficking. Platelet microthrombi and leukocyte aggregates in the pulmonary capillaries can cause microvascular obstruction, ventilation-perfusion mismatch, and decreased oxygenation. Others have shown that elevated VWF and sP-selectin levels are associated with severe COVID-19 [1, 5–13]. While previous studies have compared levels of these biomarkers in ICU patients to non-ICU patients, we here expand upon these studies by stratifying patients by severity of pulmonary disease and demonstrate that biomarkers or endothelial activation are higher in patients with more severe pulmonary disease in COVID-19 and correlated with inflammatory and vascular injury biomarkers.

## Materials and methods

### Study design and sample selection

This single-center study was conducted at The Johns Hopkins Hospital in Baltimore, MD. Patients were randomly selected from an IRB-approved, de-identified institutional COVID-19 Remnant Specimen Biorepository, consisting of samples from patients who had severe acute respiratory syndrome coronavirus 2 (SARS-CoV-2) infection confirmed by a nucleic acid test and who had excess specimen volume available after processing of clinical laboratory tests. On the date of cohort identification, the Biorepository had specimens from 232 patients. Clinical data was obtained from JH-CROWN: The COVID-19 PMAP Registry, a de-identified registry that uses the Hopkins Precision Medicine Analytics Platform and includes demographic characteristics, medical history, symptoms, vital signs, respiratory events, and laboratory results [13]. To preserve patient confidentiality, specimens and clinical data were linked by the Johns Hopkins Medicine (JHM) Core for Clinical Research Data Acquisition, part of the Johns Hopkins University Institute for Clinical and Translational Research. The study was approved by the Johns Hopkins University Institutional Review Board.

Patients were classified by maximal pre-discharge respiratory support requirement: 1) no oxygen therapy needed (No O2); 2) oxygen by nasal conventional cannula or face mask (O2 Rx); 3) high-flow O2 therapy via nasal cannula (HFNC); 4) mechanical ventilation (Vent). Patients who died (Dead) comprised a separate stratum. Plasma specimens were selected to have collection time immediately prior to the onset of maximal respiratory support or death.

### Biomarker assays

The levels of VWF antigen in blood plasma specimens were measured after a 1:100 dilution into assay diluent using a commercially available ELISA (Abcam, Cambridge, MA) performed according to manufacturer’s instructions. The levels of sP-selectin were measured after a 1:20 dilution into assay diluent using an ELISA (R&D System, Minneapolis, MN) performed according to manufacturer’s instructions. D-dimer and C-reactive protein levels were measured by the Johns Hopkins University Department of Pathology Clinical Laboratories using a serum specimen collected on the same day as the specimen used in the VWF and sP-selectin analysis. Pro-inflammatory cytokines (interferon γ (IFNγ), tumor necrosis factor α (TNFα), interleukin (IL)-1β, IL-6, IL-8, and IL-10), pro-inflammatory chemokines (CC chemokine ligand (CCL) 2, CCL4, CCL11, CCL14, CCL17, CCL24, and CXC chemokine ligand (CXCL) 10), and vascular injury biomarker (serum amyloid A (SAA), intercellular cell adhesion marker 1 (ICAM1), and vascular cell adhesion marker 1 (VCAM1)) levels were measured using multiplexed immunoassays (Meso Scale Diagnostics, Rockville, MD) performed according to manufacturer’s instructions. The following dilutions were used for each of the multiplexed assays: pro-inflammatory cytokines, 1:2; pro-inflammatory chemokines, 1:4, and vascular injury, 1:1,000.

### Statistics

Baseline patient characteristics are reported as mean (standard deviation) or number (percent). Biomarkers of interest were log-transformed to normality, and differences between groups were tested using analysis of variance with Tukey’s test for post-hoc analysis on the log-transformed variables. A p-value < 0.05 was considered statistically significant. Biomarker level correlations were determined using Spearman rank-order correlation analysis. To account for multiple comparisons, a Spearman correlation with a p-value < 0.0022 was considered statistically significant. SAS v9.4 (Cary, NC) was used for all analyses.

## Results and discussion

Remnant plasma specimens from 83 hospitalized COVID-19 patients across five disease severity groups were analyzed in this study (Table 1). The distribution of age across the groups was different, with more younger patients in the lower oxygenation groups and with more older patients in the ventilation or death groups. As expected, respiratory rates were lower in the groups with minimal oxygen requirements and higher in the groups that were mechanically ventilated or died. Additionally, the intervals between hospital admission and sample collection, and the intervals between hospital admission and maximum WHO classification were significantly higher in the Dead group. This reflects that fact that COVID-19 patients who die have experienced all the other WHO classifications during their progression to death. There were no significant differences in the prevalence of comorbidities by group. All other baseline demographics and clinical characteristics were not significantly different between the groups.

**Table 1:** Baseline demographics and clinical characteristics of the COVID-19 study cohort.

Groups	No O_2_ (N = 20)	O_2_ Rx (N = 20)	HFNC (N = 15)	Vent (N = 19)	Dead (N = 9)	*P* **
Demographic Data [N (%)]						
Age						0.008
18–39	10 (50)	9 (45)	3 (20)	4 (21)	0 (0)	
40–59	7 (35)	5 (25)	8 (53)	6 (32)	1 (11)	
60–84	3 (15)	6 (30)	4 (27)	9 (47)	8 (89)	
Gender (% Female)	10 (50)	11 (55)	8 (53)	7 (37)	4 (44)	0.83
Race						0.02
White/Caucasian	1 (5)	1 (5)	0 (0)	7 (37)	4 (44)	
Black/African American	11 (55)	10 (50)	6 (40)	6 (32)	2 (22)	
Other/Unknown	8 (40)	9 (45)	9 (60)	6 (31)	3 (33)	
Ethnicity						0.09
Not Hispanic	14 (70)	12 (60)	5 (33)	14 (74)	5 (56)	
Hispanic	6 (30)	8 (40)	10 (67)	4 (21)	3 (33)	
Unknown	0 (0)	0 (0)	0 (0)	1 (5)	1 (11)	
Obesity*						0.18
BMI < 25 kg/m^2^	5 (28)	3 (21)	0 (0)	1 (7)	3 (43)	
BMI 25–29.8 kg/ m^2^	3 (17)	4 (29)	3 (25)	7 (50)	1 (14)	
BMI ≥ 30 kg/ m^2^	10 (56)	7 (50)	9 (75)	6 (43)	3 (43)	
Pre-existing medical conditions^#^						
Coronary artery disease	1 (5)	1 (5)	0 (0)	1 (5)	2 (22)	0.28
Congestive heart failure	2 (10)	0 (0)	1 (7)	1 (5)	1 (11)	0.64
Diabetes	6 (30)	4 (20)	6 (40)	5 (26)	1 (11)	0.59
Hypertension	7 (35)	11 (55)	4 (27)	11 (58)	5 (56)	0.27
Chronic lung disease	4 (20)	4 (20)	3 (20)	4 (21)	0 (0)	0.73
None	8 (40)	9 (45)	6 (40)	6 (32)	3 (33)	0.93
Clinical Data [Median (IQR)]^						
Lymphocyte count (×10^9^/L)	1.4 (0.8, 2.0)	1.4 (0.9, 1.6)	1.0 (0.7, 1.3)	0.7 (0.4, 1.4)	0.8 (0.7, 1.3)	0.09
Pulse rate (beats/minute)	84 (71, 92)	80 (70, 98)	84 (76, 92)	86 (72, 92)	94 (80, 102)	0.62
Respiration rate (breath/minute)	18 (16, 20)	20 (18, 25)	25 (20, 33)	23 (19, 27)	33 (27, 34)	<0.001
Oxygen saturation (%)	96 (95, 99)	97 (95, 97)	96 (94, 96)	97 (95, 99)	96 (94, 97)	0.22
Interval admission to sample collection (hours)	38 (20, 64)	39 (24, 52)	51 (18, 64)	66 (45, 115)	229 (150, 316)	<0.001
Interval sample collection to WHO classification (hours)	N/A	-12 (-33, -5)	-26 (-35, -2)	-29 (-52, -13)	259 (236, 320)	<0.001

**Fisher’s exact test for categorical variables, Kruskal-Wallis test for continuous variables.

^#^Percentage totals are greater than 100% because some subjects had multiple underlying conditions.

^Clinical data were obtained on the same day that sample was collected.

*Body mass index (BMI) data were available for 18 No O_2_, 14 O_2_ Rx, 12 HFNC, 14 Vent, and 7 Dead specimens.

**Fig 1:**
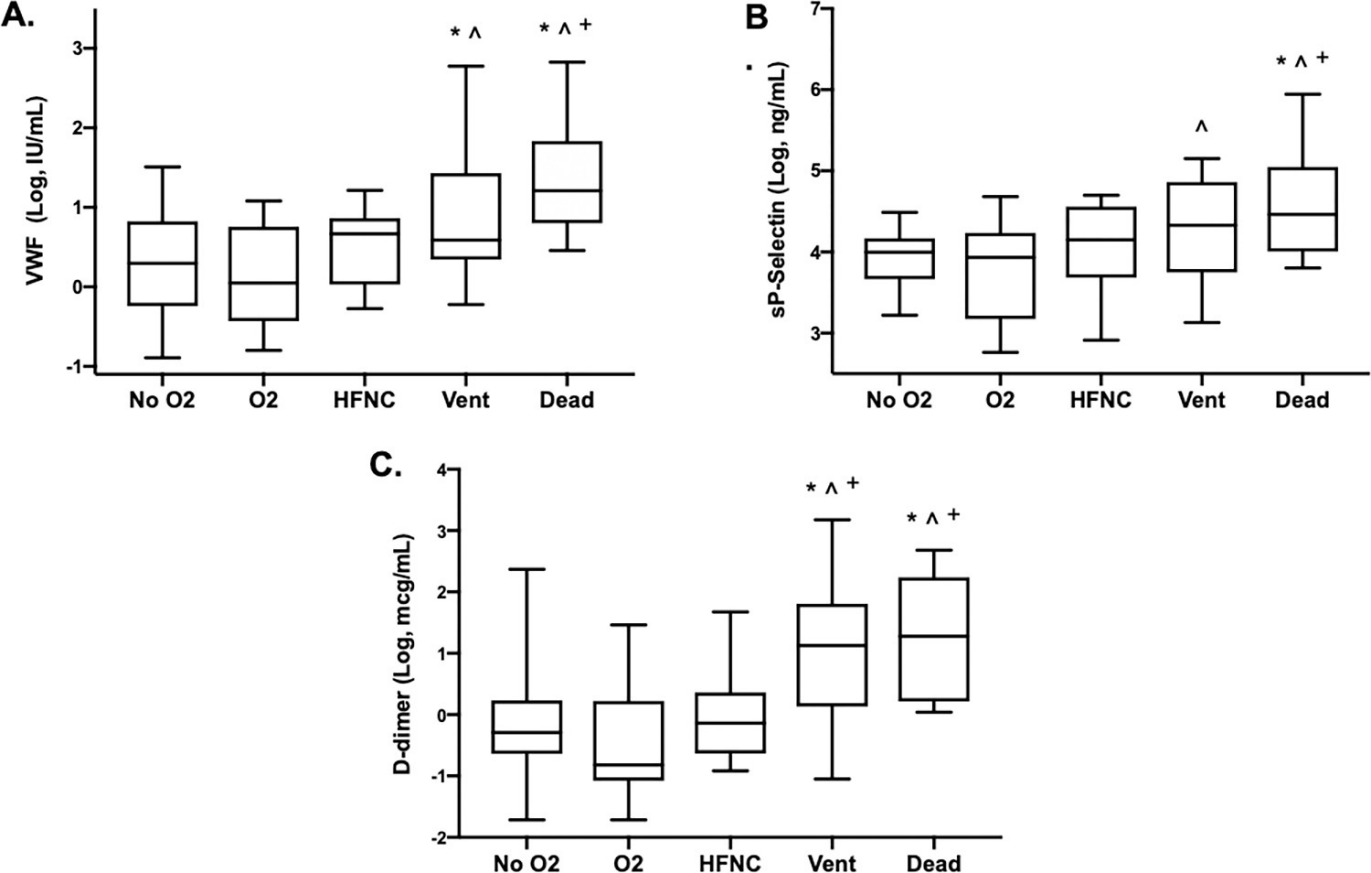
Increased severity of COVID-19 is associated with higher levels of endothelial cell activation and fibrinolysis biomarkers. The plasma levels of A) VWF, B) sP-Selectin, and C) D-dimer were measured in blood samples obtained from COVID-19 patients. Lines denote median levels with error bars representing minimum and maximum values. Sample sizes: No O2 (N = 20), O2 Rx (N = 20), HFNC (N = 15), Vent (N = 19), and Dead (N = 9). *p < 0.05 compared to No O2 group; ^*p* < 0.05 compared to O2 group; ^+^*p* < 0.05 compared to HFNC group.

We first measured the blood plasma levels of activated endothelial biomarkers across the oxygenation groups. Levels of VWF increased with severity of pulmonary disease in COVID-19 (Fig 1). VWF levels in the Vent group were higher than VWF levels in the No O_2_ group and O_2_.

### Rx group

Furthermore, VWF levels in the Dead group were significantly higher than VWF levels in the No O_2_ group or the O_2_ Rx group or the HFNC group. This finding is in agreement with earlier studies that have demonstrated that VWF levels are elevated in patients with COVID-19 [1, 7, 10–12]. Also, elevated VWF levels have been associated with clinical severity of COVID-19, particularly the risk of thrombosis, and risk of mortality from COVID-19 [1, 7, 14]. Similarly, VWF levels in patients in the ICU have previously been shown to be higher in patients in non-ICU settings [10, 12]. Additionally, one study showed that levels of VWF pro-peptide (VWFpp) are elevated in patients with COVID-19, and VWFpp levels are correlated with severity of COVID-19 [11].

Next, we measured the plasma levels of sP-selectin in the same specimens. sP-selectin levels also increased with severity of pulmonary disease in COVID-19. sP-selectin levels were higher in the Vent group than in the O_2_ Rx group (Fig 1). sP-selectin levels were higher in the Dead group than in the No O_2_ group or the O_2_ Rx group or the HFNC group. Previous studies have shown that sP-selectin levels are elevated in patients with COVID-19 [5–9]. Several studies have shown that sP-selectin levels are elevated in patients with COVID-19 in the ICU compared to sP-selectin levels of patients with COVID-19 in non-ICU settings [7, 15]. Levels of sP-selectin are associated with mortality from COVID-19 [6, 9]. Our current report extends prior studies by showing that elevated levels of sP-selectin are associated with severity of pulmonary disease in COVID-19. Taken together, these results show that higher levels of endothelial activation biomarkers VWF and sP-selectin are associated with more severe pulmonary disease in COVID-19.

In the present study, we found that levels of VWF and soluble P-selectin are elevated with increasing severity of pulmonary disease in patients hospitalized with COVID-19. VWF and sP-selectin are stored together in granules inside endothelial cells and are released together when endothelial cells are activated. While sP-Selectin is released and cleaved from the cell membrane of both activated platelets and activated endothelial cells, the primary source for secreted VWF is activated endothelial cells [16, 17]. Therefore, these data suggest that endothelial cell activation occurs during COVID-19 and sP-selectin may be a marker for severity of vascular inflammation in the lung in the setting of COVID-19.

We also measured levels of the fibrinolytic biomarker D-dimer and found that D-dimer levels were elevated in patients in the Vent and Dead groups, compared with D-dimer levels in patients in the No O_2_, O_2_ Rx, and HFNC groups (Fig 1). Elevated plasma D-dimer levels suggest that lysis of fibrin clots is occurring somewhere in the circulatory system. Previous studies have demonstrated elevated D-dimer levels in COVID-19 patients [18, 19]. The increased d-dimer levels in our subjects with increasing severity of pulmonary diseases suggests that significant fibrinolysis is occurring in these patients.

We also explored associations between biomarkers of endothelial activation and biomarkers of fibrinolysis, inflammation, and vascular injury. Interestingly, significant moderate and strong correlations between several of these biomarkers were detected. First, the levels of VWF and TNFα were positively correlated (Table 2). Since TNFα has been shown to stimulate the release of VWF in endothelial cell culture [20], it is possible that the increased levels of VWF observed in our study subjects was due to circulating TNFα.

**Table 2:** Spearman correlations* between biomarkers of endothelial cell activation, fibrinolysis, inflammation, and vascular injury.

	sPSel	CRP	D-dimer	IFNγ	IL-1B	IL-6	IL-8	IL-10	TNFα	CCL11	CCL4	CCL17	CXCL10	CCL2	CCL24	CCL13	SAA	VCAM1	ICAM1
VWF	0.14	0.26	0.21	0.14	-0.04	0.27	0.22	0.05	**0.37**	0.02	0.15	-0.13	0.23	0.30	0.04	0.05	0.14	**0.46**	0.21
	0.21	0.02	0.07	0.23	0.74	0.02	0.06	0.63	**0.001**	0.86	0.19	0.24	0.04	0.01	0.73	0.69	0.23	**<0.0001**	0.06
sPSel		0.22	**0.36**	-0.26	-0.06	0.17	0.32	-0.19	0.25	-0.07	0.16	0.01	-0.24	0.01	-0.21	0.01	0.05	-0.01	0.22
		0.06	**0.001**	0.02	0.61	0.14	0.01	0.10	0.03	0.56	0.18	0.93	0.04	0.95	0.07	0.94	0.66	0.94	0.06
CRP			0.04	0.14	0.12	**0.61**	0.31	0.28	**0.47**	0.01	0.30	-0.08	0.32	0.33	-0.01	-0.01	**0.74**	0.20	**0.51**
			0.74	0.22	0.29	**<0.0001**	0.01	0.01	**<0.0001**	0.95	0.01	0.47	0.01	0.003	0.94	0.94	**<0.0001**	0.08	**<0.0001**
D-dimer	** **			-0.02	-0.15	0.33	0.26	0.02	0.25	0.02	0.18	0.02	0.08	**0.36**	-0.07	0.13	-0.10	0.23	0.20
	** **			0.89	0.19	0.003	0.02	0.89	0.03	0.84	0.12	0.86	0.51	**0.001**	0.56	0.26	0.40	0.04	0.08
IFNγ					0.11	0.27	0.10	**0.48**	0.32	0.22	0.26	0.05	**0.67**	**0.42**	0.07	0.03	0.17	0.25	0.04
					0.34	0.02	0.40	**<0.0001**	0.004	0.06	0.02	0.69	**<0.0001**	**0.0001**	0.52	0.78	0.14	0.03	0.75
IL-1B						0.01	**0.35**	-0.08	-0.06	-0.15	-0.11	0.09	0.12	0.01	0.01	0.05	0.21	-0.05	0.03
						0.91	**0.002**	0.47	0.58	0.19	0.32	0.42	0.29	0.94	0.95	0.66	0.07	0.63	0.81
IL-6		** **					**0.38**	**0.43**	**0.42**	0.10	0.27	0.05	**0.49**	**0.53**	0.07	0.11	**0.44**	**0.37**	0.33
		** **					**0.0004**	**<0.0001**	**<0.0001**	0.39	0.02	0.68	**<0.0001**	**<0.0001**	0.53	0.33	**<0.0001**	**0.001**	0.003
IL-8					** **	** **		-0.03	**0.43**	0.05	0.12	0.11	0.16	**0.35**	0.05	**0.37**	0.17	0.13	0.10
					** **	** **		0.81	**<0.0001**	0.63	0.29	0.35	0.15	**0.002**	0.63	**0.001**	0.13	0.25	0.38
IL-10				** **		** **			**0.34**	0.14	0.33	0.08	**0.45**	0.28	0.14	-0.07	0.25	0.14	0.09
				** **		** **			**0.002**	0.21	0.003	0.47	**<0.0001**	0.01	0.22	0.56	0.03	0.21	0.45
TNFα		** **				** **	** **	** **		0.22	**0.43**	0.17	**0.34**	**0.57**	0.10	0.28	0.25	0.22	0.17
		** **				** **	** **	** **		0.05	**<0.0001**	0.12	**0.002**	**<0.0001**	0.39	0.01	0.03	0.05	0.13
CCL11											**0.44**	**0.57**	0.27	**0.47**	**0.45**	**0.49**	-0.07	0.17	-0.02
											**<0.0001**	**<0.0001**	0.02	**<0.0001**	**<0.0001**	**<0.0001**	0.54	0.13	0.85
CCL4									** **	** **		0.27	0.24	0.31	0.27	0.26	0.20	0.07	0.17
									** **	** **		0.02	0.03	0.01	0.02	0.02	0.07	0.52	0.14
CCL17										** **			0.14	0.28	**0.56**	**0.58**	-0.17	-0.13	-0.23
										** **			0.22	0.01	**<0.0001**	**<0.0001**	0.13	0.25	0.04
CXCL10				** **		** **		** **	** **					**0.70**	0.24	0.31	0.33	0.32	0.16
				** **		** **		** **	** **					**<0.0001**	0.04	0.005	0.003	0.004	0.15
CCL2			** **	** **		** **	** **		** **	** **			** **		0.23	**0.55**	0.27	**0.36**	0.26
			** **	** **		** **	** **		** **	** **			** **		0.04	**<0.0001**	0.02	**0.001**	0.02
CCL24										** **		** **				**0.49**	-0.12	0.06	-0.03
										** **		** **				**<0.0001**	0.31	0.60	0.79
CCL13							** **			** **		** **		** **	** **		0.01	0.04	0.00
							** **			** **		** **		** **	** **		0.96	0.75	0.99
SAA		** **				** **												0.19	**0.56**
		** **				** **												0.09	**<0.0001**
VCAM1						** **								** **					**0.50**
						** **								** **					**<0.0001**

* Upper value, spearman correlation coefficient; lower value, *p*-value.

Additionally, VWF levels were positively correlated with VCAM-1 levels (Table 2). This contrasted with a previous study that failed to find a significant correlation between VWF and VCAM-1 in a study population that only consisted of severely ill COVID-19 patients on mechanical ventilation [21]. Our results add to these studies by showing that VWF levels are also linked to severity of lung disease in the setting of COVID-19 inflammation.

This is the first report showing a correlation between levels of sP-selectin and D-dimer in COVID-19. Others have shown that levels of sP-selectin and D-dimer are correlated in obese children and adolescents [22]. These results suggest two possible pathways: first, cell-cell interaction mediated by P-selectin control plasmin formation, or second, that that cell-cell interactions mediated by P-selectin may be driving the generation of thrombin, which in turn leads to thrombosis, fibrinolysis, and D-dimer formation.

D-dimer levels were also positively correlated with CCL2 levels. This agrees with a previous study that demonstrated that CCL2 levels are increased in individuals with increasing D-dimer levels [23]. Interestingly, TNFα was correlated with several inflammatory modulators–CRP, CCL2, CCL4, IL-8, IL-6, and IL-10. In particular, TNFα has been shown to increase the production and release of CCL2 in several cell types [24], so it is plausible that increased TNFα was driving release of CCL2 in our subjects.

The levels of VCAM1 and CCL2 were moderately correlated in our study. Following activation by inflammatory cytokines, endothelial cells have been shown to upregulate the expression of the CCL2 [25], a pro-inflammatory chemokine that recruits innate immune cells and natural killer cells to the site of injury or infection [26]. Further, the expression of VCAM1, a cell surface adhesion molecule used by immune cells during emigration from blood to site of infection, has been shown to be upregulated by CCL2 in endothelial cells, providing an possible explanation for the observed correlation.

A strong positive correlation between CCL2 and CXCL10 was also observed. TNFα has been demonstrated to drive the development of CCL2+/CXCL10+ inflammatory macrophages in COVID-19 patients [27]. Inflammatory macrophages alter endothelial cell barrier integrity and promote microvascular thrombosis in *in vitro* models [28]. Therefore, it is possible that the inflammatory response in the patients in our study is biased towards a CCL2+/CXCL10+ inflammatory macrophage response and that these inflammatory macrophages stimulate thrombosis during COVID-19.

Our study has several limitations. First, plasma samples were not collected from the COVID-19 patients at the same time following admission to the hospital, which may introduce bias in our results. Second, the small sample sizes in our study resulted in insufficient statistical power to probe for associations between some of our biomarkers and clinical observations and performing a full sensitivity analysis of the effect of underlying conditions on our conclusions. not surprisingly the Dead group consisted of older patients. Additionally, previous studies have demonstrated that VWF levels have been shown to be higher in older patients [29]. Since VWF levels were weakly correlated with age in our study (ρ = 0.26, *p* = 0.012), our results may be confounded by this. Finally, another limitation is the timing of the collection of blood samples relative to the timing of clinical classification. The intervals between time to reach ultimate WHO classification and both sample collection and hospital admission for the Dead group were higher than all other groups, indicating that the specimens in the Dead group were collected at a later point post-admission and earlier in disease progression than in other groups. This was a function of the design of the sample collection and the fact that it takes longer for people to progress through all the WHO classification before death compared to individuals with less severe disease.

## Conclusions

In summary, we demonstrate that biomarkers of vascular endothelial cell activation and thrombosis are correlated with clinical COVID-19 pulmonary disease severity. These data suggest that endothelial activation occurs during COVID-19, although these results do not determine if endothelial activation plays an upstream role in the pathogenesis of COVID-19 or occurs downstream of other inflammatory events. One possibility is that endothelial damage is an early event in the pathogenesis of severe COVID-19, with endothelial cells releasing VWF and sP-selectin which cause microvascular obstruction of pulmonary capillaries and decreased oxygenation. The increase in endothelial cell activation biomarkers with increasing severity of disease and multiple correlations between pro-inflammatory and vascular injury biomarkers, some released by endothelial cells during inflammation, in our study suggests that SARS-CoV-2 infection activates a host inflammatory response which damages endothelial cells.

If endothelial injury is an event that occurs early in the pathogenesis of severe COVID-19, then interventions which target the vasculature may limit the progression of COVID-19. For example, therapies that antagonize the pro-inflammatory effects of sP-selectin or the pro-thrombotic effects of VWF may limit vascular inflammation and thrombosis. Novel therapies which block endothelial exocytosis may also be effective in limiting microvascular obstruction seen in severe COVID-19. Finally, further research is needed to determine which upstream mediators are responsible for damaging blood vessels and activating endothelial cells. Novel therapies targeted at the vasculature may improve the clinical outcome of patients with severe COVID-19.
